# 

**Published:** 1908-06

**Authors:** 


					﻿Texas Health Officers Association. Official Report.
Vol. XXIII.
JUNE, 1908.
-NoOII
TEXAS
Mt Bl CAL
.JOURNAL
“RED BACK”
PUBLISHED AT AUSTIN, TEXAS, BY
F. E. DANIEL, M. D.,	-	- Proprietor
PRINTED BY THE VON BOECKMA NN-JONES CO.
Subscription, $1.00 a Year, in Advance. -	Single Copies, 10 Cents
• Entered at the Postoffice at Austin, Texas, as Second-class Mail Matter
				

